# The role of serum ferritin in predicting plasma leakage among adults and children with dengue in Sri Lanka: a multicentre, prospective cohort study

**DOI:** 10.1016/j.lansea.2025.100606

**Published:** 2025-05-28

**Authors:** Chamila Mettananda, Kesara Perera, Natheeha Nayeem, Matheesha Nayanajith, Ayesha Thewage, Ranjan Premaratna, Anuradha Dassanayake, Arunasalam Pathmeswaran, Sachith Mettananda

**Affiliations:** a Department of Pharmacology, Faculty of Medicine, University of Kelaniya, Ragama, Sri Lanka; b Department of Paediatrics, Faculty of Medicine, University of Kelaniya, Ragama, Sri Lanka; c Department of Medicine, Faculty of Medicine, University of Kelaniya, Ragama, Sri Lanka; d Department of Public Health, Faculty of Medicine, University of Kelaniya, Ragama, Sri Lanka

**Keywords:** Serum ferritin, Early predictor, Dengue, Severe dengue, Plasma leakage

## Abstract

**Background:**

Early prediction of plasma leakage helps in timely management of dengue. Currently, there are no defined early predictors of plasma leakage, and identified parameters are late predictors. Raised ferritin is associated with severe dengue, but its clinical utility early in the disease to predict severe dengue is not previously reported. We studied the efficacy of day 3 or 4 serum ferritin in predicting plasma leakage among adults and children with dengue in Sri Lanka.

**Methods:**

We conducted a prospective cohort study in four hospitals in Sri Lanka from June 2022 to June 2023. Consecutive and consenting patients admitted with dengue fever were included in the study. Patients with comorbidities where ferritin could be abnormal were excluded. Serum ferritin levels were prospectively measured daily until day 8 of the illness. Physician-diagnosed plasma leakage, defined as rising haematocrit ≥ 20% from baseline or ultrasound evidence of pleural effusion or ascites, was the main outcome. Data were collected using a proforma by perusing medical records. Prediction of plasma leakage by day 3 or 4 ferritin was studied using receiver operating characteristic curves (ROC).

**Findings:**

We studied 209 patients with dengue, of which 118 (56.5%) were males and median age was 20 years (IQR 9.0–40.5). Out of 209 patients, 70 (33.5%) developed plasma leakage. Median [IQR] ferritin levels on day 3 (926 [400–2752]) and day 4 (1249 [588–3005]) in patients who developed plasma leakage were higher than ferritin levels in patients who did not develop plasma leakage (p < 0.001) (day 3: 273 [101–620]) and (day 4: 506 [220–1226]). Performance of day 3 or 4 ferritin in predicting plasma leakage showed an area under the ROC of 0.78 (95% CI 0.70–0.85). The highest serum ferritin of day 3 or 4 had a positive predictive value of 52% and a negative predictive value of 84%.

**Interpretation:**

Day 3 or 4 ferritin levels correctly predicted plasma leakage status in 78% of the study samples. Serum ferritin levels measured on day 3 or 4 can be used as an early predictor of plasma leakage in dengue.

**Funding:**

10.13039/501100011614University of Kelaniya Internal Research Grants 2022 (Grant reference number: RP/03/04/13/01/2022).

**Research in context**

**Evidence before this study**

During dengue infection, gallbladder wall oedema and an increase in haematocrit ≥ 20% from the baseline are known predictors of plasma leakage. However, the leakage process would have typically begun by the time these predictors become evident. Plasma leakage is a rapid process, usually lasting for 48 h, and needs early detection for timely action. Raised serum ferritin is identified as a prognostic marker of severe dengue, but its clinical utility early in the disease in predicting disease progression into severe dengue is not confirmed. None of the previous studies were able to develop clinically relevant cut-off values to accurately predict plasma leakage or severe dengue early in the disease course. Therefore, we studied the performance of serum ferritin done on day 3 or 4 in predicting the future likelihood of developing plasma leakage in a pragmatic study design and developed a cutoff value for clinical use.

**Added value of this study**

Our study revealed that serum ferritin levels on day 3 and 4 in patients with dengue who developed plasma leakage were higher than those of who did not develop plasma leakage. Serum ferritin levels measured early in the disease, on day 3 or 4, correctly predicted plasma leakage in 78% of patients. A serum ferritin level of 535 ng/mL or more on day 3 or 4 was associated with, and predictive of, plasma leakage (positive predictive value of 52% and negative predictive value of 84%).

**Implications of all the available evidence**

Early prediction of patients likely to progress to severe disease or dengue haemorrhagic fever helps to triage patients needing hospital admission, as only a minority develop complications. It also helps to decide on the patients who require close monitoring to detect plasma leakage early. It further helps to initiate judicious fluid management in a timely manner in order to reduce morbidity and mortality. Therefore, early prediction of plasma leakage in dengue patients will help to reduce overcrowding of wards and burnout of healthcare staff having to monitor many patients and utilise available resources cost-effectively, especially in dengue-endemic low-resource settings. Further research with multicentre studies involving many patients with a uniform case definition is needed to define the best, generalisable cutoff of ferritin to predict plasma leakage.

## Introduction

Dengue is prevalent in tropical countries. Most patients with dengue recover following a self-limiting, non-severe illness, while a small proportion progress to develop poor outcomes, characterised by plasma leakage with or without haemorrhage which is classified varyingly in two different WHO classifications as severe disease or dengue haemorrhagic fever (DHF).^1^, ^2^, ^3^ Both classifications are currently used in different epidemiological regions, but the hallmark of poor outcomes in both classifications is plasma leakage.^1^^,^^3^ Dengue is a short-lived disease and there is no specific treatment for dengue. Judicious intravascular volume management is the treatment of choice, which reduces the case fatality rate to less than 1% of severe cases.^1^

Early detection and timely fluid management is the key to the success. Therefore, early prediction of patients likely to progress to plasma leakage can be helpful in triaging patients needing hospital admission and initiating close monitoring and timely fluid management when necessary. Such triage will help to reduce overcrowding of wards during dengue epidemics and workload of healthcare staff (by decreasing the number of patients needing close monitoring facilitating the cost-effective use of available resources). Most importantly, this will help to initiate timely treatment and to reduce morbidity and mortality.

As of now, there are no early predictors of plasma leakage. Several biomarkers have been studied for early prediction of plasma leakage, but none have come into clinical management guidelines due to their limitations.^4^, ^5^, ^6^, ^7^, ^8^, ^9^ Currently used parameters like rising haematocrit and ultrasonographic evidence of gallbladder wall oedema are late features of plasma leakage.^1^ In addition, there are a few limitations of the available parameters; haematocrit can rise due to dehydration or remain unchanged due to coexistent occult bleeding even in the presence of plasma leakage, while ultrasonography is operator-dependent. Therefore, identifying reliable, freely available, low-cost, early predictors of plasma leakage in dengue is important.

Serum ferritin has been identified as a prognostic marker of dengue disease severity but its exact place as an early predictor of severe disease is not confirmed.^3^^,^^10^, ^11^, ^12^, ^13^ Ferritin levels are high in patients with severe dengue^11^^,^^14^^,^^15^ and extremely high in hemophagocytic syndrome, which is a well-known complication of dengue infection.^16^, ^17^, ^18^ A recent study reported a moderate negative correlation between serum ferritin level and platelet count and a moderate positive correlation between serum ferritin level and hospital stay.^19^ The latest systematic review and meta-analysis reported in 2023 provided a promising evidence for serum ferritin as a prognostic marker for disease severity; however, it recommended multi-centric studies involving a large number of patients with a uniform case definition to determine cut-off values to discriminate between non-severe and severe dengue.^13^ In addition, measuring ferritin levels daily would not be feasible for many patients in resource-poor, low- and middle-income countries (LMICs), especially during disease outbreaks. Hence, identifying the best time point to measure serum ferritin levels and the best cut-off level to predict patients likely to develop plasma leakage is important. We aimed to study the role of serum ferritin as an early predictor of plasma leakage, concentrating on the best timing for serum ferritin and a practical cut-off value.

## Methods

### Study design

We conducted a prospective cohort study from June 2022 to June 2023 at four centres—i.e., the University Medical and Paediatric units of Colombo North Teaching Hospital and three private hospitals—in the Gampaha District of Sri Lanka. Ethical approval for the study was obtained from the Ethics Review Committee of the Faculty of Medicine, University of Kelaniya, Sri Lanka (P/05/02/2022).

### Study participants

Consecutive, consenting patients, including adults (above 18 years of age) and children (18 years of age or below) with laboratory-confirmed dengue infection (defined as dengue NS1 antigen detection), admitted to study sites during the study period were recruited to the study until the sample size was achieved. All patients were followed up until discharge from the wards. Patients diagnosed with co-morbidities that affect serum ferritin (chronic liver diseases, connective tissue diseases, thalassaemia, iron deficiency anaemia, haematological malignancies, chronic alcohol consumers, and patients on regular or recent blood transfusions) and with co-infections (e.g., dengue-COVID-19 co-infection) were excluded. All participants were recruited after obtaining informed written consent. Informed written consent was obtained from adult patients and the parents or guardians of the children.

### Procedures

All patients were prospectively evaluated daily until discharge from the hospital. Data on demographics, symptoms, clinical features and investigations were collected prospectively by trained research assistants using a proforma. Daily serum ferritin levels were prospectively measured using VITROS® ECi chemiluminescent immunoassay until day 8 of the illness of every patient in an identified clinically accredited laboratory. Patients were carefully monitored clinically and haematologically using haematocrit to identify plasma leakage. Ultrasound examination of the abdomen and the thorax was done to confirm plasma leakage in patients with clinical or haematological suspicion of plasma leakage at any point of the disease course and the others on day 5 of the illness or admission in patients admitted after day 5. All patients received routine management as per the local guidelines except for having daily serum ferritin levels done.

### Outcomes

The main outcome of the study was the development of plasma leakage. Plasma leakage was defined as rising haematocrit ≥ 20% from baseline or ultrasound evidence of pleural effusion or ascites based on WHO guidelines.^2^^,^^3^

### Statistical analysis

The sample size was calculated for serum ferritin to detect plasma leakage with 95% sensitivity at a significant level of 95% and a desired precision of 10%^20^ using the “MKmisc” library version 1.8 in R programming language.^21^ The required minimal sample size without considering disease prevalence was 68 patients with plasma leakage (i.e., test positive). All data were entered and analysed using IBM-SPSS 28 software. A p < 0.05 was considered as statistically significant. Missing data were not imputed. Descriptive statistics were calculated separately for patients who developed plasma leakage vs. those who did not; means of normally distributed variables were compared using two-tailed *t*-test, and medians of non-normally distributed variables were compared using Mann–Whitney *U* test. The association of serum ferritin to plasma leakage was studied using binary logistic regression. The association of serum ferritin of day 3, day 4, and day 3 or 4 combined with the prediction of plasma leakage was studied separately using receiver operating characteristic curves (ROC), and the optimum cut-off points for predicting plasma leakage were calculated. The positive and negative predictive values of the selected ferritin cut-off value in predicting plasma leakage were studied. Sensitivity analyses of the predictive value of serum ferritin in adults and children were separately examined. The correlation between serum ferritin to transaminases and platelet count was assessed using the Pearson correlation coefficient as a sub-analysis.

### Role of the funding source

The funder of the study had no role in study design, data collection, data analysis, data interpretation, or writing of the report.

## Results

We studied 209 patients with confirmed dengue infection, 117 (56.0%) were adults and 92 (44.0%) were children. Of 209 patients, 118 (56.5%) were males. Baseline characteristics of the total study population are shown in Table 1, and children and adults are shown separately in Supplementary Table S1. Most patients were admitted to a hospital on day 3 or 4 of the illness.

**Table 1:** Baseline characteristics.

	Total sample (N = 209)
Male	118 (56.5%)
Age in years (median, IQR)	20 (9.0–40.5)
Age distribution	
<1 year	2 (0.9%)
1–12 years	75 (35.9%)
13–18 years	20 (9.6%)
19–64 years	109 (52.2%)
≥65 years	3 (1.4%)
BMI (mean (±SD)), kg/m^2^	22.8 (±5.27)^b^
Day of illness on admission to hospital (at enrolment)	
Day 1	11 (5.3%)
Day 2	49 (23.4%)
Day 3	69 (33.0%)
Day 4	46 (22.0%)
Day 5	23 (11.0%)
Day 6	11 (5.3%)
Medical history	
Previous dengue fever^a^	4 (1.9%)
Hypertension	22 (10.5%)
Dyslipidaemia	22 (10.5%)
Diabetes	24 (11.5%)
Fatty liver	5 (2.4%)
Long-term medications	
Antiplatelets	4 (1.9%)
Steroids	4 (1.9%)
Statins	21 (10.0%)
Antihypertensives	20 (9.6%)

Data are n (%) unless specified.

a Based on clinical history or positive serum IgG for dengue.

b Data not available for all patients.

**Fig. 1:**
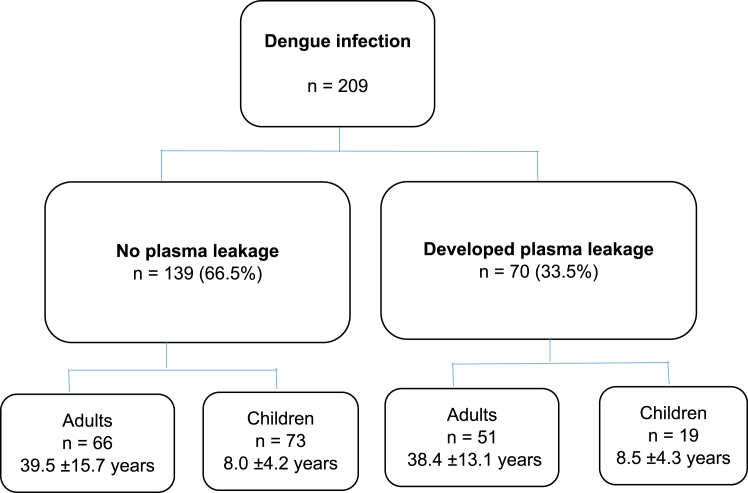
Study profile.

Of the study population, 70 (33.5%) patients (children 19/92 [20.7%] and adults 51/117 [43.6%]) developed plasma leakage (Fig. 1). The distribution of clinical and laboratory parameters during the hospital stay is shown in Table 2. Fourteen (3.8%) patients were admitted with plasma leakage. Of all, 8 (2.2%) patients were admitted to intensive care units (ICU), and 5 of them spent 3 days in ICU. A majority (137 [37.0%]) of patients stayed 4 days in hospital before discharge. There were no deaths in this cohort of patients with dengue.

**Table 2:** Distribution of laboratory parameters with the day of illness in patients who developed plasma leakage.

Day of fever	Number of patients with onset of plasma leakage diagnosed on respective dates	Haematocrit mean (SD)	Platelet count, median (IQR) x10^9^/L	AST, median (IQR) U/L	ALT, median (IQR) U/L
1	0	0	302 (194–341)	31 (8.7–52.8)	21 (21.4–21.4)
2	0	0	142 (128–178)	59 (38.8–123.8)	65 (38.8–140.5)
3	1	39.3 (6.25)	123 (77–152)	60 (36.0–108.0)	62 (33.0–128.8)
4	1	40.7 (6.14)	78.5 (45–113)	92 (46.2–185.3)	66 (39.5–191.0)
5	41	41.1 (6.17)	65.5 (31–95)	142 (66.9–235.0)	100 (59.5–219.5)
6	27	42.4 (4.67)	48 (29–77.5)	136 (62.0–257.0)	118 (53.0–231.4)
7	0	39.6 (4.94)	53 (29.5–78)	150 (100.3–276.0)	142 (68.0–263.0)
8	0	39.1 (4.87)	62 (39.5–88.0)	214 (106.7–302.0)	277 (60.0–356.5)

AST: aspartate aminotransferase; ALT: alanine aminotransferase.

**Fig. 2:**
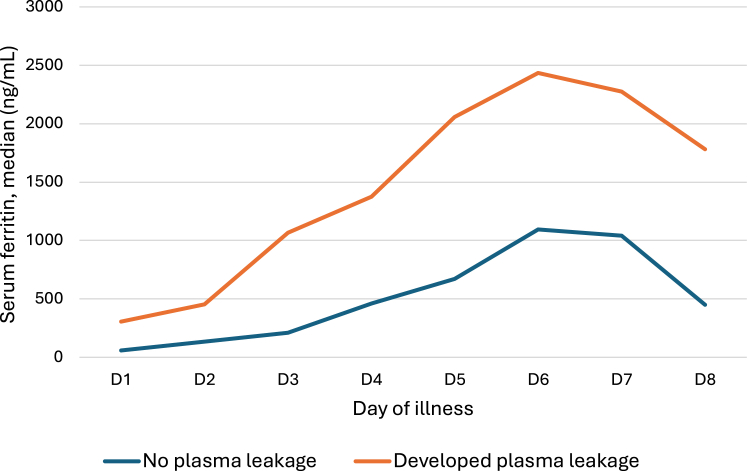
Trend of serum ferritin with time in patients who developed plasma leakage and who did not develop plasma leakage.

Serum ferritin levels increased gradually with time from the onset of illness in both groups (Fig. 2). The median serum ferritins of day 2 (p = 0.03), day 3 (p < 0.001), day 4 (p < 0.001), and day 5 were higher among patients who developed plasma leakage compared to those who did not (p < 0.001) (Fig. 2, Table 3).

**Table 3:** Daily serum ferritin levels in patients with or without plasma leakage.

Day of illness	Developed plasma leakage	No plasma leakage	p-value^a^
	Serum ferritin (ng/mL) median (IQR)	Serum ferritin (ng/mL) median (IQR)	
Day 1	306 (306–306)	67 (31–78)	0.095
Day 2	553 (179–1219)	150 (74–300)	<0.001
Day 3	926 (400–2752)	273 (101–620)	<0.001
Day 4	1249 (588–3005)	506 (220–1226)	<0.001
Day 5	1811 (1159–4456)	832 (345–2241)	<0.001
Day 6	2402 (515–5018)	1120 (421–2686)	0.005
Day 7	2220 (400–3914)	1043 (459–3349)	0.223
Day 8	1783 (100–3408)	451 (86–968)	0.384

a Mann Whitney *U* test.

Clinical and laboratory parameters associated with plasma leakage are shown in Table 4. Plasma leakage was associated with being an adult, presence of fatty liver, fever on day 4, rising serum ferritin and dropping platelet counts on days 3, 4 and 5, raising aspartate aminotransferase level (AST) and alanine aminotransferase (ALT) on day 5 (statistically significant). The sensitivity analyses in adults and children separately showed similar findings as in the total sample (Supplementary Table S2).

**Table 4:** Factors associated with plasma leakage.

	Developed plasma leakage n = 70	No plasma leakage n = 139	Odds ratio	95% CI	p-value^a^
Children, n (%)	19 (27.1%)	73 (52.5%)	0.34	0.181–0.628	<0.001
Age (median, IQR)	32 (14–46)	14 (8–37)	1.02	1.00–1.04	0.009
Male	39 (55.7%)	79 (56.8%)	1.05	0.5–1.87	0.877
Previous dengue infection	3 (4.3%)	1 (0.7%)	6.18	0.63–60.53	0.118
Diabetes	12 (17.1%)	12 (8.6%)	2.19	0.93–5.17	0.074
Hypertension	9 (12.9%)	13 (9.4%)	1.43	0.58–3.53	0.438
Fatty liver	4 (5.7%)	1 (0.7%)	8.36	0.92–76.31	0.06
Day 3 temperature ≥ 100 °F	36 (51.4%)	63 (45.3%)	1.91	0.70–5.17	0.207
Day 4 temperature ≥ 100 °F	42 (60.0%)	64 (46.0%)	2.23	1.11–4.47	0.024
Day 5 temperature ≥ 100 °F	26 (37.1%)	46 (33.1%)	1.15	0.619–2.11	0.666
Day 3 ferritin ng/mL	1067 (415–2752)	211 (92–406)	1.002	1.001–1.003	<0.001
Day 4 ferritin ng/mL	1375 (632–3924)	462 (203–1155)	1.001	1.000–1.001	<0.001
Day 5 ferritin ng/mL	2059 (1161–5015)	673 (254–2161)	1.000	1.000–1.0003	0.005
Day 3 platelet × 10^9^/L	123 (85–152)	168 (130–212)	0.99	0.98–0.99	<0.001
Day 4 platelet × 10^9^/L	79 (45–114)	142 (105–187)	0.99	0.97–0.97	<0.001
Day 5 platelet × 10^9^/L	49 (26–78)	133 (80–1610)	0.97	0.96–0.98	<0.001
Day 3 AST U/L	64 (37–135)	32 (20–56)	1.01	1.00–1.01	0.087
Day 4 AST U/L	76 (44–197)	61 (36–91)	1.00	1.00–1.01	0.195
Day 5 AST U/L	108 (62–226)	70 (39–138)	1.004	1.001–1.007	0.005
Day 3 ALT U/L	66 (38–135)	32 (20–64)	1.012	1.003–1.020	0.007
Day 4 ALT U/L	76 (44–197)	60 (36–91)	1.004	1.00–1.008	0.43
Day 5 ALT U/L	108 (62–226)	70 (39–138)	1.005	1.002–1.009	0.003

Data are n (%) or median (IQR) unless specified.

AST: serum aspartate aminotransferase; ALT: serum alanine aminotransferase.

a Unadjusted binary logistic regression.

**Fig. 3:**
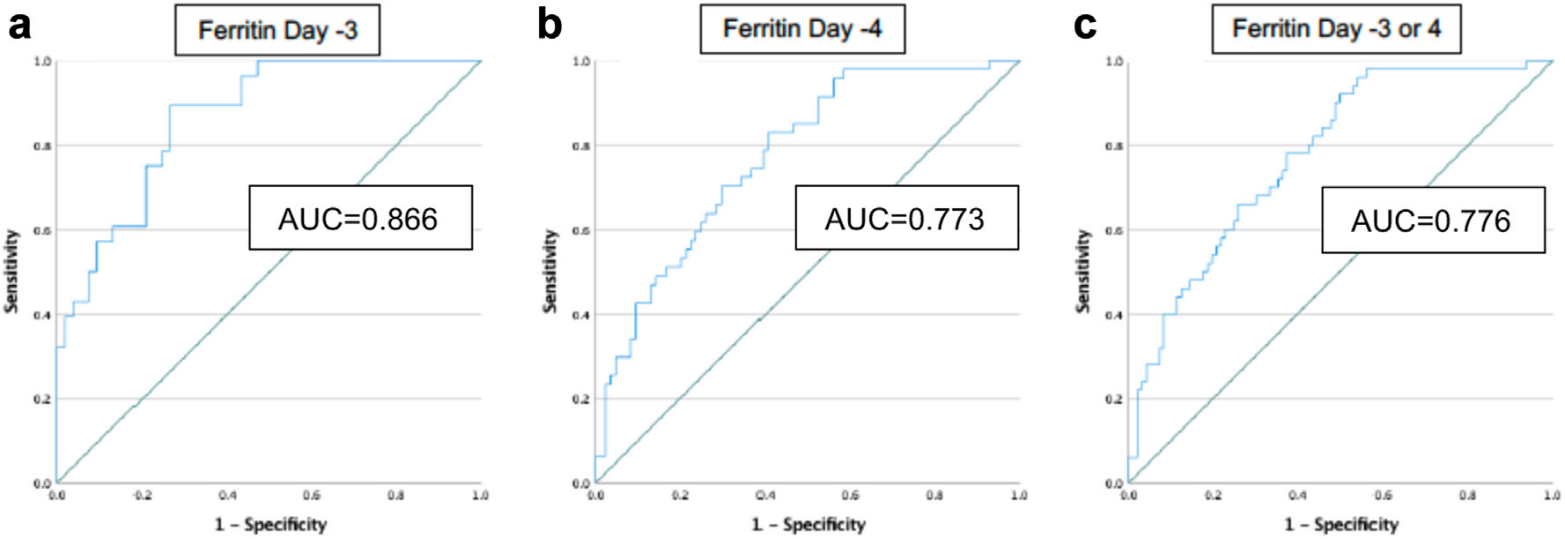
Receiver operating characteristic curves showing the performance of serum ferritin levels in predicting plasma leakage: (a) ferritin on day 3; (b) ferritin on day 4; (c) highest ferritin on day 3 or 4. AUC: Area under the ROC curve.

The predictive performance of serum ferritin done early in the disease in predicting plasma leakage is shown in Fig. 3. We studied the predictive performances of ferritin on day 3 and day 4 separately. We further studied the predictive performance of the highest ferritin of either day 3 or 4 in predicting plasma leakage separately, as the exact demarcation of days 3 and 4 is impractical in clinical settings. Serum ferritin on day 3, day 4 and highest ferritin on 3 or 4 predicted plasma leaking status with area under the curves (AUC) of 0.87 (95% CI 0.79–0.94), 0.77 (95% CI 0.69–0.85) and 0.78 (95% CI 0.70–0.85), in the ROC curves respectively (Fig. 3). As the serum ferritin levels were not normally distributed, we studied ROC curves with log-transformed serum ferritin values, which showed similar trends in the total population and when children and adults were studied separately (Supplementary Fig. S1).

We calculated the cut-off value of ferritin to predict plasma leakage using the ROC of the highest serum ferritin of day 3 or 4 (Fig. 3 C) for the cut-off to be more practically useful. A cut-off value of 535 ng/mL or more of the highest serum ferritin of day 3 or 4 had a sensitivity of 0.78 and specificity of 0.63 in predicting plasma leakage (Supplementary Table S3).

We internally validated this cutoff value in our study sample. The highest serum ferritin of day 3 or 4 of 535 ng/mL or more had a positive predictive value of 52.0% and a negative predictive value of 84.5%. The highest serum ferritin of day 3 or 4 value of 535 ng/mL or more adjusted for age and sex was associated with plasma leakage (OR 5.13, p < 0.001) (Table 5).

**Table 5:** Association of serum ferritin >535 ng/mL on day 3 or 4 in predicting plasma leakage.

	aOR	95% CI	p-value^a^
Age, years	1.02	1.00–1.04	0.10
Male sex	1.44	0.66–3.13	0.36
Highest ferritin on day 3 or 4 > 535 ng/mL	5.13	2.28–11.56	<0.001

aOR: adjusted odds ratio.

a Multivariate analysis adjusted for age and sex using binary logistic regression.

**Fig. 4:**
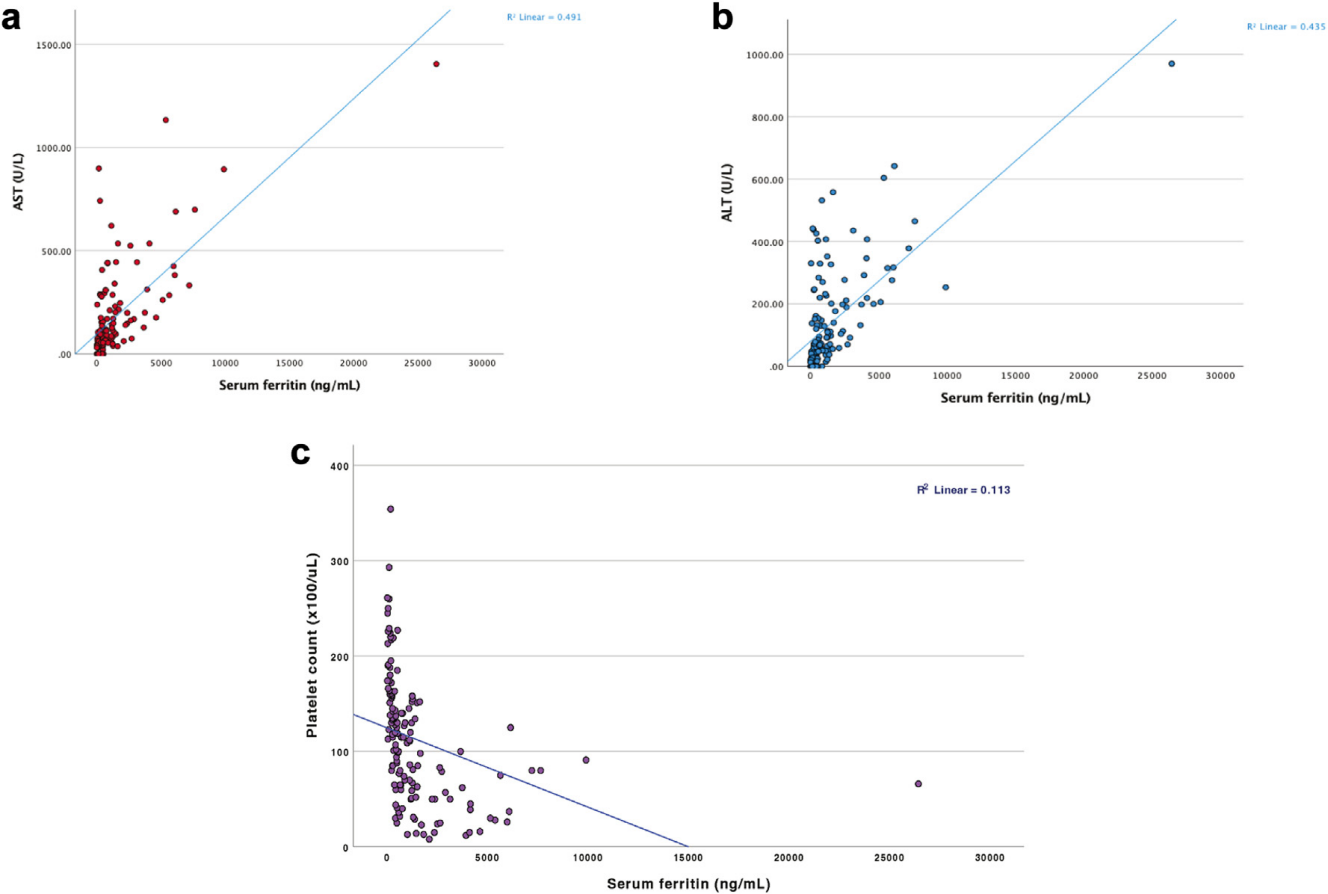
Correlation between highest serum ferritin on day 3 or 4 and day 5 and (a) AST, (b) ALT, and (c) platelet count. AST: Aspartate aminotransferase, ALT: Alanine aminotransferase.

The sub-analysis of the correlation of serum ferritin with hepatic transaminases (AST and ALT) and platelet count is shown in Fig. 4. Highest serum ferritin on day 3 or 4 showed statistically significant and moderate correlations with parameters already known to be associated with severe disease; day 5 AST (r = 0.70, 95% CI 0.60–0.78, p < 0.001), day 5 ALT (r = 0.66, 95% CI 0.56–0.74, p < 0.001) and day 5 platelet count (r = −0.33, 95% CI −0.47 to −0.18, p < 0.001).

## Discussion

We observed that day 3 and 4 serum ferritin was significantly associated with plasma leakage in both adults and children, and a serum ferritin level measured on day 3 or 4 could predict the probability of plasma leakage correctly in 78% of patients with dengue fever. A serum ferritin level of 535 ng/mL or more measured on day 3 or 4 could exclude 84.5% of patients with dengue who were unlikely to develop plasma leakage (negative predictive value). Furthermore, we observed that rising serum ferritin early in the disease significantly correlated with transaminitis manifested late in the disease. This could indicate that high ferritin in early disease also predicts impending liver involvement in dengue. The behaviour of serum ferritin was similar in children and adults when studied separately. The implications of our findings are vast. Treatment might be cost-effective as ferritin cut-off values will help to identify the patients who would require hospitalisation, close monitoring and meticulous management early in the disease. It will help to decrease the overcrowding of hospitals during epidemics and direct the limited resources available to needy patients in resource-poor settings.

Ferritin is an acute phase reactant produced by monocytes, macrophages and hepatocytes infected by the dengue virus.^22^ Ferritin is significantly elevated in dengue infection compared to other febrile illnesses except for COVID-19, which also shows a rise in ferritin levels.^22^, ^23^, ^24^ Previous evidence shows that high ferritin is associated with the severity, thrombocytopenia, elevated liver enzymes and coagulation disturbances in patients with dengue infection.^17^^,^^22^ Lodha and colleagues reported that first contact ferritin above 593 ng/mL predicted severe thrombocytopenia (<20,000/μL) with a sensitivity of 93.33%^25^ which shows very close indirect evidence for our finding of serum ferritin more than 535 ng/mL predicting plasma leakage. A small study examining the correlation between serum ferritin and severe dengue in 100 patients reported that ferritin on the day of admission is a good predictor (AUC of 0.863) and ferritin after 4 days of admission is an excellent predictor (AUC of 0.947) of severe dengue.^18^ However, this study analysed ferritin in relation to the day of admission and not to the day of illness. Furthermore, ferritin after day 4 is not an early predictor, and plasma leakage might have started in some patients by this time. Another case–control study comparing dengue fever patients and controls reported that a day 3 ferritin over 1247 ng/ml has a sensitivity of 96.4% and specificity of 91% for prediction of severe dengue, but the study design was not powered to draw this conclusion.^26^ Another study of children with dengue published in 2008 reported that plasma leak can be predicted with 81%–89% sensitivity and 36%–42% specificity with a ferritin cut-off ≥ 1200 ng/mL done on day 5–7 of the illness,^14^ which again is not an early predictor. Even though the sensitivity was high, the specificity of the cut-off value was very low. Moreover, the late time point of serum ferritin assessment precludes clinical use of this study as fluid leaking usually sets in towards day 5 of the illness. Therefore, predictive cut-offs should be developed for day 3 or 4 of the illness if they are to be clinically useful.

The association between serum ferritin and the severity of dengue illness can be explained in several ways. Ferritin is an acute-phase reactant that is increased in many infections.^11^ Ferritin level is elevated proportionately to the degree of immune activation and inflammation.^17^ The usual sources of ferritin are monocytes, macrophages, and hepatocytes, where the dengue virus thrives.^22^ Ferritin, as an acute phase reactant, depicts the level of the immune response against the dengue virus in the body.^27^ Furthermore, ferritin levels increase with major inflammation of the liver, which is a finding in severe dengue.^28^^,^^29^ Moreover, ferritin is immunogenic and leads to immune dysregulation, especially when the levels are very high, as in patients with haemophagocytic lymphohistiocytosis.^27^ Serum ferritin is extremely high in haemophagocytic syndrome, which is a well-recognised complication of severe dengue.^16^ Furthermore, very high ferritin levels are associated with increased mortality irrespective of the cause.^30^^,^^31^ A subset of patients with significantly elevated ferritin could progress to multi-organ dysfunction.^27^

Our results show that a higher proportion of adults progressed to plasma leakage compared to children, which can be explained by the pathophysiology of dengue. Plasma leakage in dengue occurs due to an exaggerated immune response to a secondary infection with a second serotype in a patient who has already developed an immune response to a previous infection with a dengue virus. First or primary infection does not lead to DHF or plasma leakage. Children are more likely to develop primary or first dengue infection, whereas adults are more likely to develop secondary infection. Therefore, adults carry a higher risk of developing DHF and plasma leakage.

There are many strengths of our study. Our objective was to develop a pragmatic cut-off value to predict plasma leakage to be used in clinical practice in LMICs. We were able to define a value with a good negative predictive value and a satisfactory positive predictive value, which is what is expected in triaging patients at the ground level. We prospectively followed up patients; therefore, information and recall biases were minimal. The primary outcome was diagnosed by an independent physician not involved in data collection. We confirmed plasma leakage using ultrasonography in all patients suspected to have plasma leakage. Our defined cutoff value is a pragmatic value to be used in a resource-poor setting without having to depend on many other variables. Defining a single cutoff value rather than using a rising titre makes this more affordable and suitable for use in LMICs.

Our study has some limitations as well. We did not study the differential associations of ferritin with dengue serotypes and viraemia. Furthermore, there was a lesser number of elderly patients enrolled in the study, and it was not powered enough to calculate separate cutoff values for children, adults, and the elderly. Also, we studied only one south Asian cohort and therefore, generalisability is low. A multicentre trial involving more patients representing other dengue endemic regions would be needed to develop more refined cutoffs, especially for adults and children, separately. In addition, external validation of the cutoff value needs to be done as a follow-up study.

In conclusion, the results of this study show that serum ferritin measured on day 3 or 4 could correctly predict plasma leakage status in 78% of the study sample. Patients with serum ferritin of 535 ng/mL or more on day 3 or 4 are five times more likely to progress to plasma leakage. This information could be used in clinical practice to decide on the indications for hospital admission in outpatient settings and the intensity of monitoring and management in inward hospitalised patients. Thus, serum ferritin done on day 3 or 4 could help to make vital management decisions, foreseeing the possibility of progression to plasma leakage. Also, serum ferritin is significantly correlated with transaminitis in dengue infection. These findings will ultimately help to reduce morbidity and mortality of patients with dengue infection by enabling cost-effective utilisation of limited resources available in hospitals in LMICs where the disease burden is at its highest.

## Contributors

Conception and design—CM, SM, AD, RP, AP; Acquisition of data—KP, NN, MN, AT.

Accessed and verified the data–CM, SM, AP; Analysis and interpretation of data CM, SM, AP; Drafting the article or revising it critically for important intellectual content—CM, SM, RP, AS, AP; Responsible for the decision to submit the manuscript—all authors; Final approval of the version to be published—all authors; Agreement to be accountable for all aspects of the work—all authors.

## Data sharing statement

Individual participant data will not be shared due to ethical restrictions. The anonymised datasets generated and analysed during the current study are available from the corresponding author upon reasonable request.

## Declaration of interests

We declare no competing interests.

## Appendix A Supplementary data

Supplementary Fig. S1 Supplementary Fig. 1. Receiver Operating Characteristic curves (ROC) showing the performance of day 3 or 4 serum ferritin (log transformed) in predicting plasma leakage: (a) in children, (b) in adults, and (c) in all. ∗age ≤ 12 years, AUC area under the curve.

Supplementary Tables S1–S3

Proposal_Ferritin to predict critical dengue disease_V2
