# Supplementary figures and images for: The role of serum ferritin in predicting plasma leakage among adults and children with dengue in Sri Lanka: a multicentre, prospective cohort study
Source: Lancet Reg Health Southeast Asia. 2025 May 28;37:100606. doi: 10.1016/j.lansea.2025.100606 (PMC12155916; doi:10.1016/j.lansea.2025.100606)

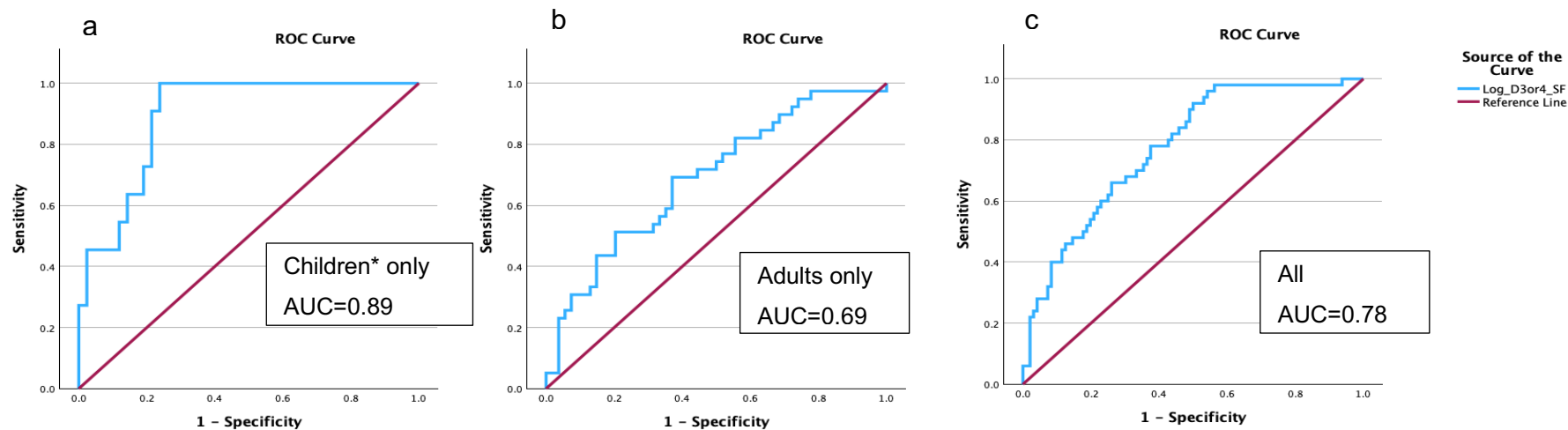

Supplementary Figure 1

Supplement: Supplementary Fig. S1 — Supplementary Fig. 1. Receiver Operating Characteristic curves (ROC) showing the performance of day 3 or 4 serum ferritin (log transformed) in predicting plasma leakage: (a) in children, (b) in adults, and (c) in all. ∗age ≤ 12 years, AUC area under the curve. [file mmc1.pdf]
