# Supplementary material for: The role of serum ferritin in predicting plasma leakage among adults and children with dengue in Sri Lanka: a multicentre, prospective cohort study
Source: Lancet Reg Health Southeast Asia. 2025 May 28;37:100606. doi: 10.1016/j.lansea.2025.100606 (PMC12155916; doi:10.1016/j.lansea.2025.100606)
Supplement: Supplementary Tables S1–S3 [file mmc2.doc]

Supplementary Table 1

Baseline characteristics of the participants; Stratified analysis in children and adults separately

|  | **Children** | **Adults** |
| --- | --- | --- |
|  | n = 92 | n = 117 |
| Age in years, mean (±SD) | 8.1(±4.2) | 39.0(±14.6) |
| Male, n (%) | 54 (58.7%) | 64 (54.7%) |
| Day of fever on admission, n |  |  |
| Day 1 | 6 | 5 |
| Day 2 | 20 | 29 |
| Day 3 | 34 | 35 |
| Day 4 | 16 | 30 |
| Day 5 | 11 | 12 |
| Day 6 | 5 | 6 |
| Previous dengue infection, n (%) | 2 (2.2%) | 2 (1.7%) |
|  |  |  |
| *Co-morbidities* |  |  |
| Hypertension, n (%) | 0 (0%) | 22 (18.8%) |
| Dyslipidaemia, n (%) | 0 (0%) | 22 (18.8%) |
| Diabetes, n (%) | 0 (0%) | 24 (20.3%) |
| Fatty liver, n (%) | 0 (0%) | 5 (4.3%) |
| BMI (kg/m^2)^, mean (±SD) | 17.2 (±5.26)^a^ | 24.4 (±4.13)^b^ |
|  |  |  |
| *Long-term medication* |  |  |
| Antiplatelets | 0 (0%) | 4 (3.4%) |
| Steroids | 2 (2.2) | 2 (1.7%) |
| Statins | 0 (0%) | 21 (17.9%) |
| Antihypertensives | 0 (0%) | 20(17.1%) |

Number; ^a^=16, ^b^=58

Supplementary Table 2

Factors associated with plasma leakage; Stratified analysis in children and adults separately

|  |  | Children |  |  | Adults |  |
| --- | --- | --- | --- | --- | --- | --- |
|  | OR | 95% CI | P* | OR | 95% CI | P* |
| Age, years | 1.03 | 0.91-1.16 | 0.617 | 1.00 | 0.97-1.02 | 0.687 |
| Male sex | 1.79 | 0.65-4.94 | 0.264 | 0.74 | 0.36-1.55 | 0.431 |
| Prior history of dengue | … |  | … | 1.3 | 0.08-21.30 | 0.854 |
| Diabetes | ….. |  | …. | 1.39 | 0.56-3.41 | 0.478 |
| Hypertension | ….. |  | …. | 0.87 | 0.34-2.24 | 0.779 |
| Fatty liver | ….. |  | …. | 5.53 | 0.60-51.10 | 0.132 |
| D3 temperature ≥100^0^F | 1.22 | 0.84-1.77 | 0.294 | **1.82** | **1.22-2.97** | **0.016** |
| D4 temperature ≥100^0^F | 1.64 | 0.70-3.83 | 0.255 | **5.27** | **1.09-25.55** | **0.039** |
| D5 temperature ≥100^0^F | 1.20 | 0.55-2.63 | 0.644 | 1.06 | 0.36-3.11 | 0.915 |
| Day 3 ferritin, ng/mL | 1.002 | 1.00-1.01 | 0.065 | **1.00** | **1.00-1.00** | **0.022** |
| Day 4 Ferritin, ng/mL | **1.001** | **1.00-1.00** | **0.018** | **1.00** | **1.00-1.00** | **0.026** |
| Day 5 Ferritin, ng/mL | 1.000 | 1.00-1.00 | 0.243 | 1.00 | **1.00-1.00** | 0.051 |
| Maximum ferritin D3 or 4, ng/mL | **1.001** | **1.0002-1.002** | **0.012** | **1.0003** | **1.00-1.001** | **0.022** |
| Day 3 plateletx10^9^/L | 0.989 | 0.977-1.001 | 0.075 | **0.988** | **0.978-0.998** | **0.015** |
| Day 4 platelet x10^9^/L | **0.965** | **0.945-0.985** | **<0.001** | **0.982** | **0.973-0.991** | **<0.001** |
| Day 5 platelet x10^9^/L | **0.948** | **0.923-0.974** | **<0.001** | **0.980** | **0.972-0.989** | **<0.001** |
| Day 3 AST, U/L | 1.006 | 1.00-1.01 | 0.059 | 1.01 | 1.00-1.02 | 0.997 |
| Day 4 AST, U/L | 0.999 | 0.99 -1.00 | 0.679 | 1.01 | 1.00-1.01 | 0.064 |
| Day 5 AST, U/L | 1.002 | 1.00-1.01 | 0.478 | **1.00** | 1.00-1.01 | 0.013 |
| Day 3 ALT, U/L | **1.014** | **1.000-1.027** | **0.045** | **1.013** | **(1.004-1.022)** | **0.005** |
| Day 4 ALT, U/L | 0.998 | 0.991-1.006 | 0.651 | **1.005** | **(1.001-1.008)** | **0.006** |
| Day 5 ALT, U/L | **1.012** | **1.002-1.022** | **0.022** | **1.004** | **(1.001-1.006)** | **0.003** |

* unadjusted binary logistic regression

AST - serum aspartate aminotransferase, ALT - serum alanine aminotransferase

Supplementary Table 3

Coordinates of the Receiver Operating Characteristic curve of ferritin on day 3 or 4 in predicting plasma leakage

|  | | |
| --- | --- | --- |
| Positive if Greater Than or Equal To | Sensitivity | 1-Specificity |
| 12.63 | 1 | 1 |
| 13.82 | 1 | 0.99 |
| 20.06 | 1 | 0.979 |
| 29.21 | 1 | 0.969 |
| 36.34 | 1 | 0.958 |
| 42.68 | 1 | 0.948 |
| 47.99 | 1 | 0.938 |
| 53.39 | 0.98 | 0.938 |
| 56.49 | 0.98 | 0.927 |
| 60.77 | 0.98 | 0.917 |
| 72.22 | 0.98 | 0.906 |
| 83.31 | 0.98 | 0.896 |
| 93.75 | 0.98 | 0.885 |
| 102.6 | 0.98 | 0.865 |
| 104.61 | 0.98 | 0.854 |
| 107.51 | 0.98 | 0.844 |
| 122.5 | 0.98 | 0.833 |
| 137.05 | 0.98 | 0.823 |
| 140.84 | 0.98 | 0.813 |
| 146.28 | 0.98 | 0.802 |
| 150.5 | 0.98 | 0.792 |
| 155.5 | 0.98 | 0.781 |
| 165.5 | 0.98 | 0.771 |
| 172.73 | 0.98 | 0.76 |
| 178.48 | 0.98 | 0.75 |
| 185.1 | 0.98 | 0.74 |
| 195 | 0.98 | 0.729 |
| 203.41 | 0.98 | 0.719 |
| 208.96 | 0.98 | 0.708 |
| 215.2 | 0.98 | 0.698 |
| 219.5 | 0.98 | 0.688 |
| 223 | 0.98 | 0.677 |
| 224.5 | 0.98 | 0.667 |
| 226.5 | 0.98 | 0.656 |
| 241.75 | 0.98 | 0.646 |
| 257.25 | 0.98 | 0.635 |
| 260.66 | 0.98 | 0.625 |
| 263.36 | 0.98 | 0.615 |
| 266.67 | 0.98 | 0.604 |
| 269.42 | 0.98 | 0.594 |
| 270.45 | 0.98 | 0.583 |
| 275.2 | 0.98 | 0.573 |
| 282.67 | 0.98 | 0.563 |
| 286.85 | 0.96 | 0.563 |
| 298.58 | 0.96 | 0.552 |
| 319.92 | 0.96 | 0.542 |
| 334.83 | 0.94 | 0.542 |
| 342.6 | 0.94 | 0.531 |
| 355.5 | 0.92 | 0.531 |
| 369.16 | 0.92 | 0.521 |
| 379.31 | 0.92 | 0.51 |
| 392.2 | 0.92 | 0.5 |
| 400.15 | 0.9 | 0.5 |
| 405 | 0.9 | 0.49 |
| 413.77 | 0.88 | 0.49 |
| 421.8 | 0.86 | 0.49 |
| 425.88 | 0.86 | 0.479 |
| 432.24 | 0.84 | 0.479 |
| 440.89 | 0.84 | 0.469 |
| 454.6 | 0.84 | 0.458 |
| 472.5 | 0.82 | 0.458 |
| 480.85 | 0.82 | 0.448 |
| 482.85 | 0.82 | 0.438 |
| 489 | 0.8 | 0.438 |
| 494.46 | 0.8 | 0.427 |
| 500.66 | 0.78 | 0.427 |
| 508.39 | 0.78 | 0.417 |
| 510.91 | 0.78 | 0.406 |
| 515.73 | 0.78 | 0.396 |
| 521.95 | 0.78 | 0.385 |
| 535.65 | 0.78 | 0.375 |
| 556.65 | 0.76 | 0.375 |
| 571.95 | 0.74 | 0.375 |
| 583.4 | 0.74 | 0.365 |
| 596.1 | 0.72 | 0.365 |
| 618.05 | 0.72 | 0.354 |
| 637.85 | 0.7 | 0.354 |
| 645.5 | 0.7 | 0.344 |
| 670 | 0.7 | 0.333 |
| 692.5 | 0.68 | 0.333 |
| 700.45 | 0.68 | 0.323 |
| 712.45 | 0.68 | 0.313 |
| 738.5 | 0.68 | 0.302 |
| 768 | 0.66 | 0.302 |
| 790.05 | 0.66 | 0.292 |
| 821.55 | 0.66 | 0.281 |
| 842.75 | 0.66 | 0.271 |
| 863.85 | 0.66 | 0.26 |
| 888 | 0.64 | 0.26 |
| 947.4 | 0.62 | 0.26 |
| 1005.9 | 0.62 | 0.25 |
| 1050.4 | 0.6 | 0.25 |
| 1102 | 0.6 | 0.24 |
| 1117 | 0.6 | 0.229 |
| 1126 | 0.58 | 0.229 |
| 1134 | 0.58 | 0.219 |
| 1148.5 | 0.56 | 0.219 |
| 1173.65 | 0.56 | 0.208 |
| 1203.65 | 0.54 | 0.208 |
| 1224 | 0.54 | 0.198 |
| 1228.1 | 0.52 | 0.198 |
| 1236.6 | 0.52 | 0.188 |
| 1247.2 | 0.5 | 0.188 |
| 1253.2 | 0.5 | 0.177 |
| 1261.5 | 0.48 | 0.177 |
| 1274.5 | 0.48 | 0.167 |
| 1290.5 | 0.48 | 0.156 |
| 1337.5 | 0.48 | 0.146 |
| 1383.5 | 0.46 | 0.146 |
| 1412 | 0.46 | 0.135 |
| 1442 | 0.46 | 0.125 |
| 1470 | 0.44 | 0.125 |
| 1494.95 | 0.44 | 0.115 |
| 1514.45 | 0.42 | 0.115 |
| 1578 | 0.4 | 0.115 |
| 1642 | 0.4 | 0.104 |
| 1679.95 | 0.4 | 0.094 |
| 1762.25 | 0.4 | 0.083 |
| 1959.8 | 0.38 | 0.083 |
| 2168.5 | 0.36 | 0.083 |
| 2294.5 | 0.34 | 0.083 |
| 2362.5 | 0.32 | 0.083 |
| 2437 | 0.32 | 0.073 |
| 2560 | 0.3 | 0.073 |
| 2629.5 | 0.28 | 0.073 |
| 2680 | 0.28 | 0.063 |
| 2810.5 | 0.28 | 0.052 |
| 3017.5 | 0.28 | 0.042 |
| 3397 | 0.26 | 0.042 |
| 3700 | 0.24 | 0.042 |
| 3832.21 | 0.24 | 0.031 |
| 4014.41 | 0.22 | 0.031 |
| 4123.7 | 0.22 | 0.021 |
| 4145.99 | 0.2 | 0.021 |
| 4384.44 | 0.18 | 0.021 |
| 4878.06 | 0.16 | 0.021 |
| 5255.61 | 0.14 | 0.021 |
| 5508.5 | 0.12 | 0.021 |
| 5808.5 | 0.1 | 0.021 |
| 6025 | 0.08 | 0.021 |
| 6110.7 | 0.06 | 0.021 |
| 6672.2 | 0.06 | 0.01 |
| 7417.5 | 0.06 | 0 |
| 8766.55 | 0.04 | 0 |
| 18160.55 | 0.02 | 0 |
| 26426 | 0 | 0 |
