# Supplementary material for: The role of serum ferritin in predicting plasma leakage among adults and children with dengue in Sri Lanka: a multicentre, prospective cohort study
Source: Lancet Reg Health Southeast Asia. 2025 May 28;37:100606. doi: 10.1016/j.lansea.2025.100606 (PMC12155916; doi:10.1016/j.lansea.2025.100606)
Supplement: Proposal_Ferritin to predict critical dengue disease_V2 [file mmc3.docx]

**Place of serum ferritin done in early dengue as a predictor of disease progression to critical illness**

|  | Introduction | Serum ferritin done in early dengue has been shown a promising predictor of progression to severe illness but has not yet been included in local or international guidelines. There are no studies highlighting the value of serum ferritin in this context among dengue patients in Sri Lanka. |
| --- | --- | --- |
|  | Objective | 1. To assess serial serum ferritin levels in dengue illness at its different phases 2. To study the association of serum ferritin levels done in early dengue to critical illness 3. To estimate the sensitivity and specificity of serum ferritin in detecting critical illness in patients diagnosed with dengue fever (DF) |
|  | Study design | Observational study |
|  | Study setting | Two private hospitals in Gampaha district and professorial medical and paediatric units of Colombo North Teaching Hospital |
|  | Study population | All eligible adults and children with confirmed dengue fever who consent to participate in the study |
|  | Definition of intervention | No intervention. Serum ferritin levels will be studied on D2, D3, D4, D5, of the illness |
|  | Sample size | 68 patients with dengue - critical illness and 68 patients with dengue fever without critical illness |
|  | Sampling method | Consecutive, consenting patients with DF will be recruited to the study until the required sample size is achieved.  DF is defined by having fever and other symptoms of dengue with a positive NS1 antigen test.  Critical illness in dengue is defined as dengue infection with evidence of fluid leakage measured by   - Rising haematocrit of 20% or more together with - Evidence of intravascular fluid leakage (presence of pleural effusion or ascites) |
|  | Data collection method | Interviewer administered questionnaire. Clinical and laboratory parameters of the patients will be collected from the BHT. Serum ferritin level will be done on routine blood samples taken for usual care of dengue patients. |
|  | Main ethical issues | All participants will have the study explained in detail. Informed written consent/proxy consent will be obtained from all participants/ parents of children who are willing to participate in the study. Permission from Director, NCTH and the private hospitals will be obtained. The usual care of patients will not be changed/compromised at any point. There is no additional cost or extra venepunctures to the patient. The cost for the serum ferritin will be borne by the study team. Data confidentiality will be maintained. Information gathered will be disseminated through publication only. |

**Introduction and literature survey**

Dengue illness has a spectrum of clinical manifestations and intravascular fluid leakage leads to the development of critical illness. Undetected critical illness carries a high mortality. Therefore, it is very important to detect patients who are likely to develop critical illness early in order to prevent morbidity and mortality. Although several markers have been studied(1-4) to predict the development of critical illness early in the disease, there are currently no clinical or laboratory indicators with good sensitivity and specificity that can predict progression to critical illness. Furthermore, currently available markers are not freely available, clinically useful and affordable especially in developing countries (5, 6).

Several studies have demonstrated that ferritin is significantly elevated in dengue infection than in other febrile illnesses (7) but with exception of COVID 19. Serum ferritin is known to be associated with severity and prognosis in dengue illness (8). There are three main reasons postulated for this observation. First is that ferritin is an acute-phase protein that is known to increase in many infections including dengue (7). Ferritin is produced by monocytes, macrophages and by hepatocytes, which are infected by the dengue virus(9). Ferritin level is elevated proportionately with the degree of immune activation and inflammation. Increased ferritin levels represent an important host defence mechanism that deprives bacterial growth, protects immune cell function, limit the production of free radicals and mediates immunomodulation(10). The second is that ferritin levels increase with significant inflammation of the liver of any cause; ie; hepatitis (11, 12). Third is that ferritin itself is immunogenic and is a key mediator of immune dysregulation, especially in extreme hyperferritinemia, via direct immune-suppressive and pro-inflammatory effects(10). Elevated ferritin levels are associated with an increase in mortality irrespective of the source of elevation (13, 14). A subset of patients with significantly elevated ferritin could progress to multi-organ dysfunction. Hyper-ferritinaemia is significantly associated with thrombocytopenia, elevated liver enzymes and coagulation disturbances in patients with dengue fever(9). Furthermore, serum ferritin is extremely high in hemophagocytic syndrome (HPS) which is a well-known complication of dengue haemorrhagic fever(15).

Ferritin has been studied as a predictor of critical dengue disease in a few studies but has not yet come to local or international guidelines (16-18). Serum ferritin more than 1291 ng/ml is reported 82.6% sensitive and 100% specific in differentiating dengue from other fevers (7). It is significantly higher in patients with the critical disease compared to simple dengue fever (19). Serum ferritin 1247 ng/ml on day 3 has a sensitivity of 96.4% and specificity of 91% for prediction of progression to critical illness (20). It is reported that high serum ferritin on the day of admission could predict the disease severity with 76.9% sensitivity and 83.3% specificity (21), while day 4 ferritin is an excellent marker of critical disease (22).  Available evidence suggests that measuring ferritin levels in patients with dengue may be very useful in the following circumstances.

1. Around Day 4 of fever

2. When the patient is clinically unwell

3. When the liver function tests are significantly deranged

**Justification and aims**

Ferritin could be a good predictor of critical dengue disease but has not been included in dengue management guidelines yet. Ferritin level may offer information regarding the degree of immune activation and inflammation which can provide additional information to the physician to predict the progression of DF to critical illness early in the course of the disease. Although it may not be feasible to get routine serum ferritin in all patients with DF, identification of the pattern of rising in ferritin in dengue patients and detecting the best timing to do ferritin levels would be most cost-effective for low/middle-income countries like Sri Lanka. However, there are no reports of its predictive value in dengue patients in Sri Lanka.

Therefore, we aim to study the value of serum ferritin level as a predictor of dengue fever (DF) progression to critical illness.

**Objectives**

1. To study serial serum ferritin levels in dengue illness at its different phases
2. To study the correlation of serum ferritin levels done in early dengue to critical illness
3. To study the sensitivity and specificity of serum ferritin in predicting critical illness in patients diagnosed with dengue fever (DF)

**Methodology**

**Study design -**

Prospective observational study

**Study setting -**

Professorial medical and paediatric units of North Colombo Teaching Hospital, Ragama and Two private hospitals in Gampaha district

**Duration of the study -**

12 months

**Study population -**

adults and children with confirmed dengue fever admitted to the study settings

**Sample size and calculation –**

The sample size was calculated to detect 95% sensitivity for serum ferritin to detect dengue critical disease considering the below assumptions (23);

Assumptions:

- Sensitivity of the test = 95%
- Minimal acceptable sensitivity of the test = 85%
- Significance level = 95%
- Desired precision = 10%
- Required sample size without considering disease prevalence
  - DF patients (i.e. test negative) = 68
  - DHF patients (i.e. test positive) = 68

Sample size calculation was done using “MKmisc” library version 1.8 in R programming language(24).

Inclusion criteria

All eligible adults and children with confirmed dengue fever ie: Dengue NS1 antigen were admitted to the study facilities over the study period.

(Any patient 18 years or above in age is defined as an adult

Any patient less than 18 years of age is defined as a child)

Exclusion criteria

1. Patients not consenting to the study

2. Patients with other illnesses where ferritin level could be not normal

Chronic liver diseases

Connective tissue diseases

Chronic alcohol consumers

Patients with haemoglobin less than 10g/dL

Thalassaemia patients or patients on regular/recent blood transfusions

Patients with haematological malignancies

3. Patients with co-infections (ie: Dengue-Covid co-infections)

Patient recruitment -

Consecutive, consenting adult patients and children with parental/guardian assent who are eligible with DF will be recruited to the study until the required sample size is achieved.

DF is defined by having fever and symptoms compatible with dengue with a positive NS1 antigen test.

The critical disease is defined according to WHO guidelines as dengue infection (17) with evidence of fluid leakage measured by

• Rising haematocrit of 20% or more together with

• Evidence of pleural effusion or ascites

Consecutive dengue fever patients admitted to the study facilities will be recruited. Clinical and laboratory data of all patients will be collected by trained medical graduates to a proforma (Annex 1). A serum ferritin level of all patients will be measured on Day 2,3,4 & 5 of the illness on a 2 ml blood sample that is routinely drawn for clinical management of these patients. The serum of the sample will be separated and stored in a -20 C freezer at the Thalassaemia lab at the Faculty of Medicine, Ragama, and will be transported to the identified laboratory. The samples will not be stored for any future studies and will be discarded after serum ferritin analysis.

**Data analysis**

Initially, correlation analysis will be carried out between serum ferritin levels at different time points in dengue illness. Subsequently, logistic regression analysis will be carried out to evaluate the presence of critical illness and serum ferritin at different time points. The models will be adjusted for possible confounders. Generalized additive models will be used to assess non-linear associations in exposure variables. Separate longitudinal data analysis will be carried out to evaluate the association between dengue critical illness and serum ferritin at different time points. Overdispersion of the models will be assessed and hierarchical models will be adopted as necessary. The optimum cut off points of serum ferritin levels to detect dengue critical illness will be determined using ROC curves. The sensitivity and specificity of a specific day’s serum ferritin level in the prediction of dengue critical illness will be studied. The significance level will be set at p <0.05.

**Dissemination of study findings**

Findings will be published in peer-reviewed journals and will be presented at medical conferences locally and internationally.

**Budget**

|  | **Description** | **Total (Rs.)** |
| --- | --- | --- |
| 1. **Consumables** | Printing questionnaires, transport, gloves etc. | **40,000.00** |
| 1. **Sample analysis if outsourced** | Rs 1000 (per test) x 560 tests | **Rs. 560,000.00** |
| **Grand Total** |  | **600,000.00** |

Funding will be sought from the Faculty of Medicine, Research grants 2022 (Rs 500,000/=) . We have already presented this to the faculty and is now being reviewed by an external examiner. The remaining money around Rs 100,000/= will be borne by the investigators.

**Ethical issues**

**Assessment of risks/benefits**

This is not an interventional study and there is no change in the standard of care and is associated with no risks to the patients. There is no extra cost or blood tests to the patients. Serum ferritin levels will be done on a 2 ml blood drawn for routine daily blood tests in the management of dengue fever. The findings of the study could be beneficial widely.

**Procedure for obtaining consent**

The participants/ parents of the children will be informed and explained about the study including the data collection procedure and the blood tests. Informed written consent of Adult patients/assent of the parents/guardians of the children will be obtained. The patients will be made aware of the ability to withdraw consent at any point without having to give reasons. Costs for serum ferritin levels will be borne by external funding assigned for the project. Permission will be obtained from the directors of the North Colombo Teaching Hospital, the two private hospitals and the consultants in charge of the patients at the study settings.

**Justification for including vulnerable population**

These patients are not a vulnerable group of patients.

**Procedures to protect the rights of participants**

Confidentiality/Privacy

All the data will be kept confidential. Personal identification details will not be shared.

Voluntary participation right to refuse or withdraw without penalty

The participants will be informed that they have the right to decide whether to participate in the study and to withdraw from the study at any point.

Responsibilities of the researchers

The researchers will be responsible to protect the rights of the study subjects on all occasions.

Providing psychological and medical support to patients

This is an observational study. The findings of ferritin reports will not be informed to the patient unless the patient insists on as this is not a part of the standard of care of dengue patients.

**Gant chart**

| Activity | 2022 | | | | | | | | | | | |  |  |
| --- | --- | --- | --- | --- | --- | --- | --- | --- | --- | --- | --- | --- | --- | --- |
|  | Jan | Feb | Mar | Apr | May | Jun | July | Aug | Sep | Oct | Nov | Dec | Jan | Feb |
| Literature survey |  |  |  |  |  |  |  |  |  |  |  |  |  |  |
| Proposal writing |  |  |  |  |  |  |  |  |  |  |  |  |  |  |
| Ethical clearance |  |  |  |  |  |  |  |  |  |  |  |  |  |  |
| Data collection |  |  |  |  |  |  |  |  |  |  |  |  |  |  |
| Data entry |  |  |  |  |  |  |  |  |  |  |  |  |  |  |
| Data analysis |  |  |  |  |  |  |  |  |  |  |  |  |  |  |
| Report writing |  |  |  |  |  |  |  |  |  |  |  |  |  |  |

1. Kularatnam GAM, Jasinge E, Gunasena S, Samaranayake D, Senanayake MP, Wickramasinghe VP. Evaluation of biochemical and haematological changes in dengue fever and dengue hemorrhagic fever in Sri Lankan children: a prospective follow up study. BMC Pediatrics. 2019;19(1):87.

2. Pawitan JA. Dengue virus infection: predictors for severe dengue. Acta Med Indones. 2011;43(2):129-35.

3. Tsheten T, Clements ACA, Gray DJ, Adhikary RK, Furuya-Kanamori L, Wangdi K. Clinical predictors of severe dengue: a systematic review and meta-analysis. Infectious Diseases of Poverty. 2021;10(1):123.

4. Hegazi MA, Bakarman MA, Alahmadi TS, Butt NS, Alqahtani AM, Aljedaani BS, et al. Risk Factors and Predictors of Severe Dengue in Saudi Population in Jeddah, Western Saudi Arabia: A Retrospective Study. The American Journal of Tropical Medicine and Hygiene. 2020;102(3):613-21.

5. Srikiatkhachorn A, Green S. Markers of dengue disease severity. Curr Top Microbiol Immunol. 2010;338:67-82.

6. Sangkaew S, Ming D, Boonyasiri A, Honeyford K, Kalayanarooj S, Yacoub S, et al. Risk predictors of progression to severe disease during the febrile phase of dengue: a systematic review and meta-analysis. Lancet Infect Dis. 2021;21(7):1014-26.

7. Roy Chaudhuri S, Bhattacharya S, Chakraborty M, Bhattacharjee K. Serum Ferritin: A Backstage Weapon in Diagnosis of Dengue Fever. Interdisciplinary Perspectives on Infectious Diseases. 2017;2017:7463489.

8. Valero N, Mosquera J, Torres M, Duran A, Velastegui M, Reyes J, et al. Increased serum ferritin and interleukin-18 levels in children with dengue. Braz J Microbiol. 2019;50(3):649-56.

9. van de Weg CAM, Huits RMHG, Pannuti CS, Brouns RM, van den Berg RWA, van den Ham H-J, et al. Hyperferritinaemia in dengue virus infected patients is associated with immune activation and coagulation disturbances. PLoS Negl Trop Dis. 2014;8(10):e3214-e.

10. Kernan KF, Carcillo JA. Hyperferritinemia and inflammation. Int Immunol. 2017;29(9):401-9.

11. Chen Q, Gao M, Yang H, Mei L, Zhong R, Han P, et al. Serum ferritin levels are associated with advanced liver fibrosis in treatment-naive autoimmune hepatitis. BMC Gastroenterology. 2022;22(1):23.

12. Batsaikhan B, Gantumur G, Huang CI, Yeh ML, Huang CF, Lin ZY, et al. Elevated serum ferritin level associated with hepatic steatosis and fibrosis in hepatitis C virus-infected patients. J Chin Med Assoc. 2019;82(2):99-104.

13. Bennett TD, Hayward KN, Farris RW, Ringold S, Wallace CA, Brogan TV. Very high serum ferritin levels are associated with increased mortality and critical care in pediatric patients. Pediatr Crit Care Med. 2011;12(6):e233-6.

14. Kadoglou NPE, Biddulph JP, Rafnsson SB, Trivella M, Nihoyannopoulos P, Demakakos P. The association of ferritin with cardiovascular and all-cause mortality in community-dwellers: The English longitudinal study of ageing. PloS one. 2017;12(6):e0178994-e.

15. Yamashita S, Furukawa NE, Matsunaga T, Hirakawa Y, Tago M, Yamashita S-I. Extremely High Serum Ferritin: An Instrumental Marker of Masquerading Adult-Onset Still's Disease with Hemophagocytic Syndrome. Am J Case Rep. 2017;18:1296-301.

16. WHO. Dengue and severe dengue 2021 [cited 2021 02.10.2021]. Available from: <https://www.who.int/health-topics/dengue-and-severe-dengue#tab=tab_3>.

17. World Health Organization. Regional Office for South-East A. Comprehensive Guideline for Prevention and Control of Dengue and Dengue Haemorrhagic Fever. Revised and expanded edition. New Delhi: WHO Regional Office for South-East Asia; 2011.

18. WHO, editor. Dengue: guidelines for diagnosis, treatment, prevention and control -- New edition. India: WHO; 2012.

19. Ab-Rahman HA, Rahim H, AbuBakar S, Wong P-F. Macrophage Activation Syndrome-Associated Markers in Severe Dengue. International Journal of Medical Sciences. 2016;13(3):179-86.

20. Murmu AR, R M. Correlation between serum ferritin level and severity of dengue fever in a tertiary care center: an observational study. International Journal of Research in Medical Sciences; Vol 9, No 6 (2021): June 2021. 2021.

21. Soundravally R, Agieshkumar B, Daisy M, Sherin J, Cleetus CC. Ferritin levels predict severe dengue. Infection. 2015;43(1):13-9.

22. Suresh SC, Hanumanthaiah R, Ramakrishna C, Sandeep R, Narasimhasetty PS, Ramakrishna V, et al. Serum Ferritin As a Prognostic Indicator in Adult Dengue Patients. The American Journal of Tropical Medicine and Hygiene. 2021;104(3):1072-8.

23. Flahault A, Cadilhac M, Thomas G. Sample size calculation should be performed for design accuracy in diagnostic test studies. J Clin Epidemiol. 2005;58(8):859-62.

24. MKmisc: Miscellaneous functions from M. Kohl. R package version 1.8 [Internet]. 2021. Available from: <https://www.stamats.de>.
